# Knowledge-Driven Manufacturing Process Innovation: A Case Study on Problem Solving in Micro-Turbine Machining

**DOI:** 10.3390/mi12111357

**Published:** 2021-11-03

**Authors:** Dong Zhang, Gangfeng Wang, Yupeng Xin, Xiaolin Shi, Richard Evans, Biao Guo, Pu Huang

**Affiliations:** 1 Key Laboratory of Road Construction Technology and Equipment of MOE, School of Construction Machinery, Chang’an University, Xi’an 710064, China; 2019125089@chd.edu.cn (D.Z.); 2020225023@chd.edu.cn (P.H.); 2 College of Mechanical and Vehicle Engineering, Taiyuan University of Technology, Taiyuan 030024, China; xinyupeng@tyut.edu.cn; 3 School of Mechanical Engineering, Northwestern Polytechnical University, Xi’an 710072, China; sxl86@mail.nwpu.edu.cn; 4 Faculty of Computer Science, Dalhousie University, Halifax, NS B3H 4R2, Canada; R.Evans@dal.ca; 5 China Huayin Ordnance Test Center, Huayin 714200, China; guo_biao1234@126.com

**Keywords:** micromachining, micro-turbine, computer-aided innovation, innovation design, smart manufacturing, knowledge-based engineering, problem solving

## Abstract

Micromachining techniques have been applied widely to many industrial sectors, including aerospace, automotive, and precision instruments. However, due to their high-precision machining requirements, and the knowledge-intensive characteristics of miniaturized parts, complex manufacturing process problems often hinder production. To solve these problems, a systematic scheme for structured micromachining process problem solving and an innovation support system is required. This paper presents a knowledge-based holistic framework that enables process planners to achieve micromachining innovation design. By analyzing innovation design procedures and available knowledge sources, an open multi-source Machining Process Innovation Knowledge (MPIK) acquisition paradigm is presented, including knowledge units and a knowledge network. Further, a MPIK network-driven structured process problem-solving and heuristic innovation design method was explored. Subsequently, a knowledge-driven heuristic design system for machining process innovation was integrated in the Computer-Aided Process Innovation (CAPI) platform. Finally, a case study involving specific process problem-solving and innovation scheme design for micro-turbine machining was studied to validate the proposed approach.

## 1. Introduction

Advancements in artificial intelligence, advanced manufacturing, automation control, and optics and microelectronics technologies, have evolved the way in which industrial products are designed, with emphasis now being placed on miniaturization and structural complexity [1,2,3,4,5]. Product miniaturization reduces overall weight and material and energy consumption, and improves space utilization, but places greater requirements on parts micromachining technology [6,7]. In the field of modern manufacturing technologies, micromachining refers to the application of precision or ultra-precision cutting tools to remove metal, composite, alloy, ceramics, and other engineering materials, to obtain microscale parts or structures using mechanical force. The geometric size of such micro-parts or structures is often at the centimeter level or smaller, creating difficulties in the conventional fixing and clamping of parts. Due to high-precision requirements and the small size of parts, they can easily deform, especially during the machining of thin-walled structures, micro-holes, and slender shafts [6,8].

Extant research into micromachining technology has predominantly focused on micromachining tools, cutting tools, and the influence of micromachining parameters on machining quality and efficiency. Takacs et al. [9] used fine-grained carbide end mills to conduct experimental research into micromilling for metallic materials. To overcome the nonlinearity of the conventional servomechanism, Wang et al. [10] developed a new control system for three-axis ultra-precision micromilling using coreless linear motors and air bearing slides. Scholars have also optimized the microcutting process parameters and process routes of specific materials or parts. To improve the micro-surface roughness of aluminum alloy, Cardoso and Davim [11] explored the optimization of cutting parameters such as feed rate and machining strategies. In the machining of micro-parts and structures, it is necessary to optimize process planning, not only to ensure machining accuracy and efficiency, but also to control the feed rate and chip thickness to avoid deformation. Son et al. [12] studied the relationship between the friction of a tool-workpiece and the minimum cutting thickness in ultra-precision diamond micro-cutting, and developed a corresponding ultra-precision cutting model. In light of the machining process problems associated with complex parts, several process innovation technologies, and methods for microparts with specific structures have been developed. To avoid tool breakage and to ensure the geometrical accuracy of the machined feature in micromachining, Ba et al. [13] proposed a cutting force prediction method that combined sensitivity analysis and finite element simulation. To solve the contradiction between minimum chip thickness and relatively large tool deformations, under the condition of micromachining, Balazs et al. [14] produced a dynamic milling tool path strategy and performed systematic experiments.

However, the influencing factors and formation mechanism of micromachining process problems are relatively complicated, and effective process innovation and optimization schemes are difficult to design structurally. Most existing innovations are achieved through trial-and-error or unstructured brainstorming sessions, which lead to micromachining process innovations relying on the inspiration of only a few designers, leading to instability in innovations and resource wastage [15,16,17]. The structured approach of heuristic innovation has distinct characteristics from other traditional problem-solving methods, such as trial-and-error and brainstorming, which usually attempt to directly find specific factual solutions for factual problems.

Computer-Aided Innovation (CAI) technology has provided an effective means to assist designers in obtaining innovation inspiration and in improving efficiency during technological innovation. CAI adopts computer technology, combined with modern design methodologies, innovation thinking theory, knowledge management, information and communication technology, cognitive psychology, and other multidisciplinary fields [18]. To further shorten the product development lifecycle, against the background of CAI, Cugini et al. [19] proposed an integrated CAI methodology to ensure the interoperability of multiple systems by adopting optimization systems as a bridge between CAI and Product Lifecycle Management (PLM) systems. Xu et al. [20] studied knowledge management and its application in product innovation design, and proposed an integrated knowledge modeling and management approach for continuous innovation. Esterhuizen et al. [21] studied the role of knowledge management in enhancing innovation design and identified a knowledge creation path as a critical enabler for innovation capability maturity. To enhance computer-aided problem solving, Duran-Novoa et al. [22] presented a strategy that incorporated dialectical negation operators in evolutionary algorithms and TRIZ (The Theory of Inventive Problem Solving) principles [23]. Similarly, Cakir and Cilsal [24] built a TRIZ-like contradiction matrix-based access system, with a corresponding knowledge base that can provide design recommendations for turning, milling, and drilling processes. Delgado-Maciel et al. [25] proposed a methodology that combines TRIZ tools with system dynamics simulations to speed up new products development and to identify key inventive problems to ensure technical feasibility. By analyzing technological developments in open innovation and Web 2.0, Hüsig and Kohn [26] proposed an Open CAI 2.0 paradigm, based on Closed CAI 1.0. Flores et al. [27] introduced a conceptual framework that combined collective intelligence and a logical TRIZ approach for the inventive problem resolution of Open CAI 2.0.

The aforementioned studies have completed valuable exploratory work into aspects of innovation design methods, theoretical model construction, and system tool development, but have mainly focused on product innovation design rather than manufacturing process innovation. In addition, existing computer-aided tools, such as Computer-Aided Process Planning (CAPP) and PLM, have mainly been used to improve efficiency and the standardization of process planning and management [28,29] rather than to solve manufacturing process problems structurally and create or improve process methods; therefore, they cannot systematically improve the level of manufacturing process research and the development of enterprises [15,30,31]. In considering the intentions and application objects of existing studies, and the fact that the proposed design systems and methods cannot be directly applied to the manufacturing process innovation of conceptual designs, a Computer-Aided Process Innovation (CAPI) methodology and knowledge accumulation framework was proposed in previous research [32]. Whilst CAI is mostly employed in the field of product innovation design, some specific achievements demonstrate that knowledge-driven computer-supported process innovation tools can effectively inspire and guide designers to implement structured process problem solving [24,33,34,35]. At present, studies into CAPI are at the early stages of development and, therefore, the theory and method of manufacturing process innovation design, as well as knowledge management and applications supporting the innovation design process, need to be systematically explored [32,36,37].

This paper examines how to effectively acquire and organize machining innovation knowledge and how to apply this knowledge to facilitate heuristic process problem-solving and innovation scheme design. By analyzing the innovation design procedure and knowledge sources, several types of process innovation knowledge units are formally represented. Then, an open multi-source Machining Process Innovation Knowledge (MPIK) acquisition paradigm is proposed, and a heuristic machining process problem-solving method, based on innovation knowledge, is explored. Further, a Knowledge-driven Heuristic Design System for Machining Process Innovation (MPI-KHDS) is constructed to support knowledge-driven micro-turbine machining process innovation.

The rest of the paper is organized as follows: In Section 2, a holistic framework of knowledge-based manufacturing process innovation design is introduced. Section 3 presents the innovation knowledge acquisition and management of machining processes, including knowledge units and knowledge networks. Section 4 describes the knowledge network-driven machining process problem-solving and innovation design method. Then, the specific process of problem solving and the innovation design of micro-turbine machining is implemented in Section 5 with the support of the MPI-KHDS prototype system. Finally, conclusions of this study are summarized in Section 6.

## 2. A Holistic Framework of Knowledge-Based Manufacturing Process Innovation Design

**Figure 1:**
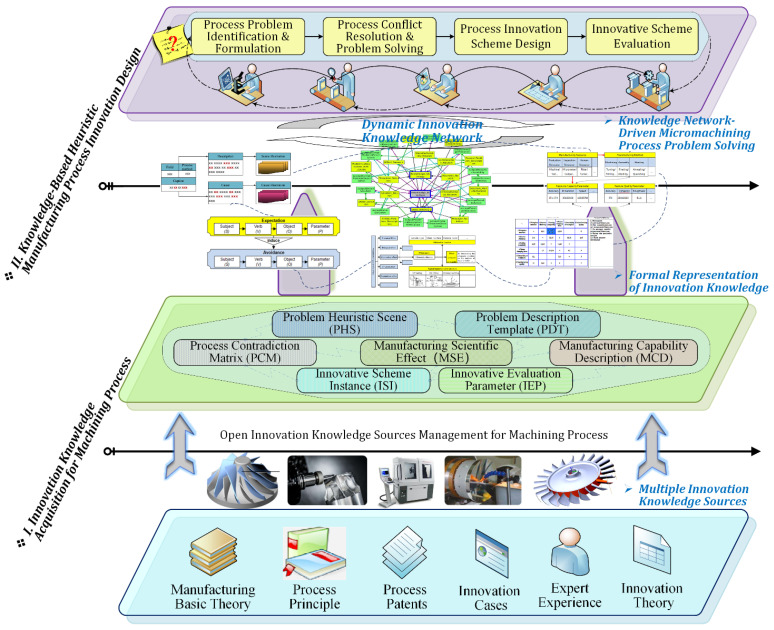
A holistic framework of knowledge-based heuristic manufacturing process innovation.

Computer-aided manufacturing process innovation is a knowledge-based design procedure whereby formal process knowledge is derived from multiple manufacturing sources. The knowledge of these sources must be effectively acquired, organized, and formalized into manufacturing process innovation knowledge that can support the corresponding design stages of CAPI. As shown in Figure 1, this paper proposes a holistic framework of a knowledge-based manufacturing process innovation design, which includes two stages of innovation knowledge acquisition and formal expression, and a knowledge-based heuristic manufacturing process innovation design. In considering that MPIK has the characteristics of wide-range dispersion, strong fuzziness, and high experience, an open innovation knowledge acquisition method for the machining process is required in Stage I, which can effectively manage both explicit and implicit knowledge sources. Thus, MPIK can be organized according to the whole procedure of machining process innovation design. In Stage II, on the basis of formal innovation knowledge representation, different types of innovation knowledge units need to be further associated and organized, and a dynamic innovation knowledge network should be constructed to inspire process designers to effectively carry out structured innovation design in complex manufacturing process problem-solving scenarios.

## 3. Innovation Knowledge Acquisition and Management for Machining Process

### 3.1. Sources and Contents of Machining Process Innovation Knowledge

MPIK is the basis of machining process problem solving in the context of computer-aided innovation. High-quality solutions depend on massive process innovation knowledge. Through the analysis of the computer-aided machining innovation design process and the required knowledge content, this paper divides the sources of process innovation knowledge as follows:

(1) Basic manufacturing theories. Basic manufacturing theories refer to the general laws of natural science that can provide guidance for applied research. They are the basic scientific principles that manufacturing process innovation activities should follow, such as the theories and principles of physics, chemistry, geometry, materials, and biology.
(2) Technical process principles. Technical process principles relate to the manufacturing methods and mechanisms that guide the production of parts and entire products in the manufacturing environment, such as cutting and measuring.
(3) Process patents and innovation cases. Process patents are an important knowledge source for manufacturing innovation that reflect the latest advances in multi-disciplinary fields involved in new technologies, new processes, new methods, etc. Innovation cases can reflect the manufacturing ability and characteristics of an organization and can be used as a powerful reference for solving similar process problems and innovative scheme design.
(4) Expert experience. Expert experience refers to machining know-how, and experience and methods in the field of manufacturing that exist in the minds of manufacturing professionals and operators. Expert experience is a valuable asset accumulated through project experience, so explicit expert experience can be regarded as an important innovation knowledge source for the machining process.
(5) Innovation theories. Innovation theories refer to methodologies that use available resources to create something new, such as TRIZ and OTSM (General Theory of Powerful Thinking) [38,39]. Such theories can provide guidance and assistance for the implementation of manufacturing process innovation.

According to the analysis in Section 2, the procedure of process innovation design can be split into four steps that include: process problem identification and formulation, process conflict resolution and problem solving, process innovation scheme design, and innovative scheme evaluation. The purpose of the innovation process is to identify contradictions in problems and resolve them with dialectical thinking. The Theory of Inventive Problem Solving (TRIZ) follows this approach and receives widespread attention due to its structured procedure of problem solving. In the process problem identification step, typical problem scenarios have heuristic features and can help technicians to improve the efficiency and quality of problem recognition. In the innovative scheme evaluation step, the feasibility of innovative solutions require evaluation according to the evolution law of technology systems and the manufacturing capacity of the corresponding enterprise.

Different types of MPIK must be applied to the corresponding innovation design stage. Thus, MPIK can be divided into the following types to support the entire process of innovation design: Problem Heuristic Scene (PHS), Problem Description Template (PDT), Process Contradiction Matrix (PCM), Manufacturing Scientific Effect (MSE), Innovative Scheme Instance (ISI), Innovative Evaluation Parameter (IEP), and Manufacturing Capability Description (MCD). Table 1 shows the concept and function of each type of MPIK.

### 3.2. Open Multi-Source Knowledge Acquisition for Machining Process Innovation

Manufacturing experts and technicians possess a powerful ability to solve machining problems, although the discrete and unstructured knowledge cannot be used directly in machining process innovation. Hence, the process of MPIK acquisition should recognize that knowledge can be turned from tacit to explicit, from discrete to associative, and from rough to refined. Considering dynamic knowledge forms, the organizing process of MPIK can be divided into three phases: discrete knowledge, Process Innovation Knowledge Unit (PIKU), and process innovation knowledge network.

The process innovation knowledge network is similar to a biological neural network. Neural networks consist of large numbers of neurons, accept external stimuli, and output a control action through the interaction between neurons. The process innovation knowledge network contains a large number of PIKUs, accepts the stimulus of process problems, and outputs innovative solutions through the interaction between PIKUs. Due to their similarity, we propose a construction method of the process innovation knowledge network by imitating a neural network. Thus, the PIKU, which should have specific interfaces and accurate parameters, can be considered a process innovation knowledge neuron in the knowledge network, and an adequate number of PIKUs constitute a Process Innovation Knowledge Neural Network (PIKNN) in the environment of machining process problem solving.

**Figure 2:**
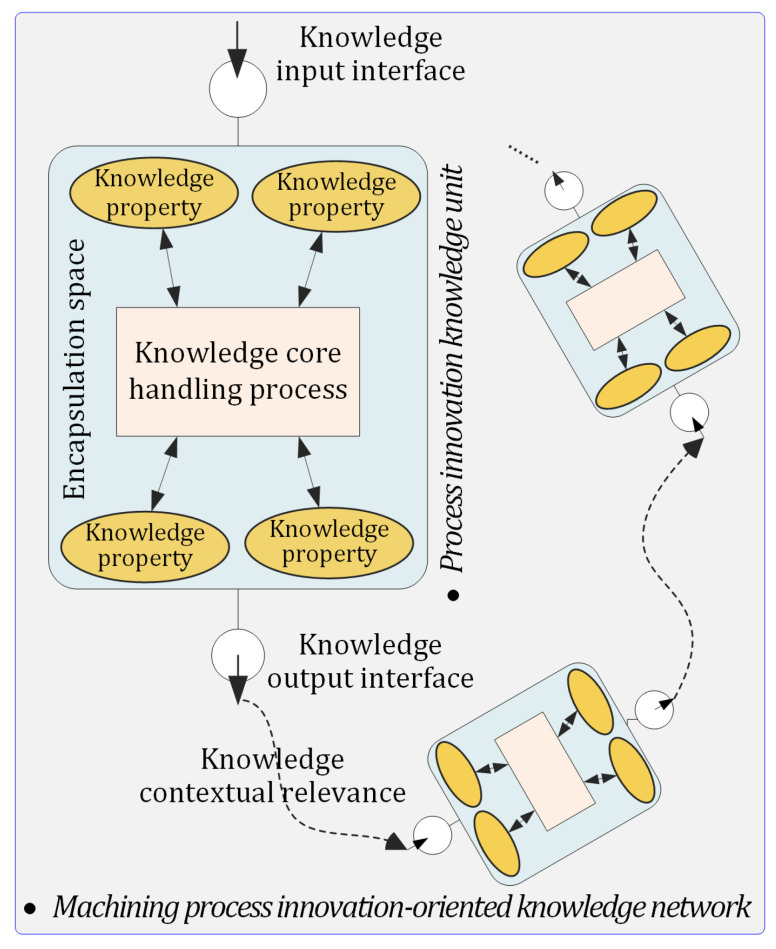
Illustration of the machining process innovation-oriented knowledge network.

The PIKU has a knowledge parameter as an input interface and a knowledge result as an output interface, which corresponds to the dendrite and axon of a neuron, respectively. The encapsulation space of a PIKU is mapped to the cell membrane of a neuron. The knowledge attribute is mapped to the cytoplasm of a neuron. The core handling process is mapped to the nucleus of a neuron. The hierarchical structure of the machining process innovation-oriented knowledge network is shown in Figure 2 and formally defined as follows.

**Definition** **1.** *A manufacturing process innovation-oriented knowledge network is a set of spatial knowledge structures, formally represented as*

(1) $$MPIK_{\Omega }=\left\{KU,\;CTR,\;U\right\}$$

*where*KU*is a set of multi-type machining process innovation knowledge units,*CTR*is a set of knowledge contextual relevance for specific machining process innovation scenarios, and*U*is a set of process innovation participants*.

**Definition** **2.** *A MPIK unit has the ability to solve certain types of machining process problems and deliver information. It is denoted as*

(2) $$KU=\langle P,\;I_{I},\;I_{O},\;E,\;U\rangle$$

*where* P *is a set of knowledge properties,* $I_{I}$ *and* $I_{O}$ *are the sets of knowledge input interfaces and knowledge output interfaces, respectively;* E *represents an encapsulation space for the whole knowledge unit. Several types of machining process innovation knowledge,* $\Pi_{KU}=\left\{PHS,PDT,PCM,MSE,ISI,IEP,MCD\right\}$ *, are mainly applied in the machining process innovation design.*

**Definition** **3.** *The knowledge contextual relevance of machining process innovation is further denoted by*

(3) $$CTR=\left\{\langle ku,k,r,k^{\prime },u\rangle |ku\in KU,k,r,k^{\prime }\in O,u\in U\right\}$$

*where*$k,\;r,\;and\;k^{\prime }$*are ontological entities defined in the machining process innovation domain ontology*O*, and*r*stands for a contextual relationship between*k*and*$k^{\prime }$.

**Definition** **4.** *Ontology*O*consists of a series of concepts and relationships that represent domain knowledge models. It can be represented as*

(4) $$O:=\left(C,R,{ℰ}_{R},I_{C}\right)$$

*where*C*and*R*represent a set of classes and a set of relations, respectively;*${ℰ}_{R}\subseteq C\times C$*is a set of relationships between classes, which can be denoted as a set of triples*$\left\{\langle c,r,c^{\prime }\rangle |c,c^{\prime }\in C,r\in R\right\}$*; and*$I_{C}$*is a power set of instance sets of a class*c∈C.

The PIKUs can be divided into several categories, which can be built as knowledge ontologies in accordance with the corresponding knowledge classifications. Meanwhile, the process data ontologies must be built to provide input parameters and output results for the knowledge interfaces. Table 2 describes the basic process data ontologies and the knowledge ontologies in the machining process innovation domain. The semantic relationships between process data ontologies and knowledge ontologies characterize the knowledge interfaces.

**Figure 3:**
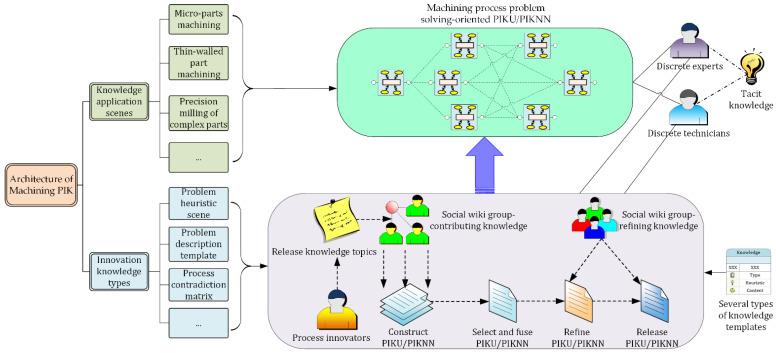
Open knowledge acquisition paradigm for machining process innovation.

Social network technologies, based on relationship networks and interested topics, can provide an exchanging, sharing, and manifesting knowledge platform beyond background and specialty. Wiki technologies can provide a knowledge refining and associating platform through page locking and collaborative editing [32]. Therefore, for the knowledge building stages of PIKU and PIKNN, combining the characteristics of social networks with wikis, an open MPIK acquisition paradigm, based on a social-wiki network, was constructed and is presented in Figure 3.

From the perspective of knowledge contributors, social wikis can quickly establish innovative communities and seek appropriate participants for knowledge acquisition. From the perspective of MPIK, social wikis can make knowledge contributors participate in the collaborative editing of various innovation knowledge and guarantee the refinement of the knowledge obtained. Meanwhile, the PIKU social wiki network can collect personal discrete knowledge and refine and store it in a public knowledge space, while the PIKNN social wiki network can make technicians collaboratively add knowledge relevance for PIKUs in order to form a dynamic, self-organizing machining process problem-solving-oriented PIKNN.

## 4. Knowledge-Driven Problem-Solving and Innovation Design Method for Micromachining Process

### 4.1. Structured Problem-Solving and Innovation Design Procedure of Machining Process

The structured problem solving of machining process innovation can be regarded as a dialectical process from concrete to abstract to concrete again. First, a specific and factual process problem is reduced to its essential state and recorded in a conceptual format. In its conceptual form, the problem can be matched with one or more conceptual solutions. Then, the identified conceptual solution can be transformed into a specific, factual solution that can answer the original factual problem. In this way, heuristic process innovation, using computer-aided technology, can reduce inherent mindset and knowledge limitations [22,40], essentially identifying problems and providing possible heuristic principles or new solutions for the machining process innovators.

**Figure 4:**
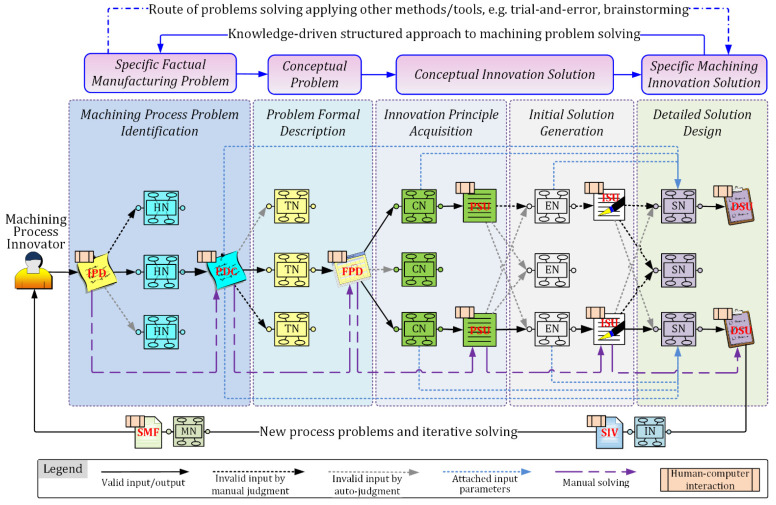
Structured problem-solving and innovation design procedure of machining process.

The basic procedure for structured heuristic process problem solving is shown in Figure 4 and described as follows.

(1) Process problem identification. The machining process innovator manually inputs the IPD according to the identified machining problem. Then, the HNs that meet the threshold of semantic similarity with the description of IPD are automatically listed for heuristic innovation. The innovator can then interactively input the cause of the process problem and generate the PDC, based on the selected HNs. Finally, the selected HNs transfer the PDC to all TNs.
(2) Problem formal description. After the TNs accept the PDC, they will determine if they can be applied to the PDC according to the problem domain. Then, the innovator can retrieve the objective and obstacles of the problem, based on the PDC and recommended TNs, and interactively input them to form the FPD, which will be transferred to all CNs. Finally, the FPD formation process is constrained and standardized using the domain ontology database.
(3) Innovation principle acquisition. Each CN represents a unit of the machining contradiction matrix. After accepting the FPD, the CNs automatically parse the contradiction parameters in the FPD. If the parsed contradiction parameters are matched with the CNs, the solving principles in the CNs will be extracted as PSU under human–computer interaction and output as the function requirement format to all ENs.
(4) Initial solution generation. Each EN is a triple combination of effect, function, and typical structure. After accepting the PSU, each EN will automatically build a comparison between the function requirement of PSU and its realizable function. If they are matched, the corresponding effect and typical structure will be output as ISU to the SNs.
(5) Detailed solution design. When the SN has accepted the ISU, it calculates the similarity between itself and the ISU, referencing the previous TN, CN, and EN. Then, the system automatically lists the SNs that meet the threshold of similarity for the machining process innovator to refer to. The innovator can then reuse the SNs or interactively input information to generate the DSU inspired by the SNs.

It should be noted that, after the detailed solution design of machining process innovation is completed, the innovativeness and manufacturability of the scheme need to be further evaluated with the INs and MNs in order to meet the actual manufacturing conditions of the specific organization. In addition, the specific problem solving of machining process innovation design may result in new process problems; thus, iterative problem solving is often required during the entire innovation process.

### 4.2. Heuristic Innovation Knowledge Processing Architecture of Problem Solving

Many discrete PIKUs with semantic relationships can constitute a static knowledge network. After accepting the input stimulation of machining process problems, the static knowledge network selects the appropriate PIKUs to form a dynamic temporary knowledge network. Then, the knowledge signal will be transmitted to the semantic knowledge network, and the process problem will be solved gradually. The basic rules for machining process problem solving with PIKUs are listed in Table 3.

**Figure 5:**
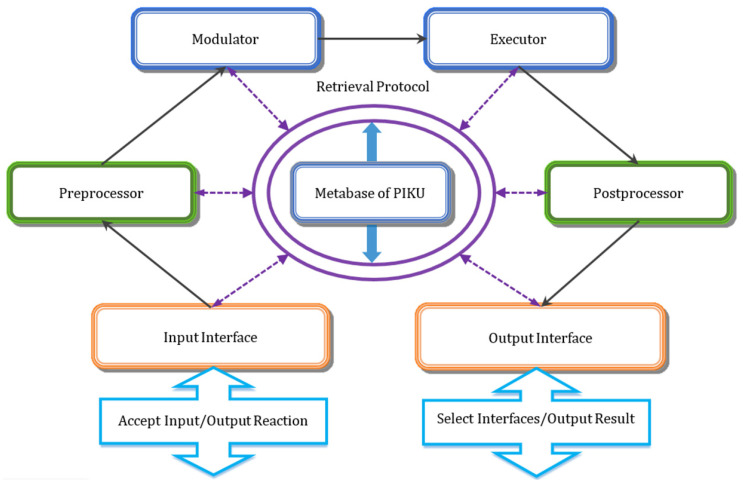
Knowledge processing architecture of machining PIKU.

For complex process problems, there may exist multiple contradictions. In such situations, the problem usually needs to be split into multiple single and simple problems. If the innovative solution leads to new machining process problems, the solving process must be iteratively executed until the contradictions are completely resolved or a compromise is reached. The continuous processing of PIKUs for process problems constitute the process of problem solving and innovation design. Thus, the core of machining process problem solving lies in the knowledge processing of each PIKU as input data. In fact, the knowledge processing of innovation design is a cognitive and reasoning process of PIKUs, and can be regarded as an Integrated Neuro-Cognitive Architecture (INCA) [41]. If the problem is solved through the INCA, it will be clearer and more reasonable, and complies with the innovative thinking process of the innovators. The knowledge processing architecture of the PIKU simulating the INCA for the input problem is shown in Figure 5.

The architecture of knowledge processing for machining process innovation consists of seven parts. The input interface that simulates the function of the thalamus accepts the input parameters and feeds-back the decision on whether to process or not. The output interface that simulates the function of the motor cortex selects all suitable subsequent PIKUs and transfers the processing result to them. The preprocessor simulating the function of the hippocampus makes pretreatments for the input parameters, for example, extracting contradiction units from the FPD of a machining process problem. The postprocessor that simulates the function of the cerebellum ganglia creates the posttreatments for the processing result in order to meet the formal requirements of the output information, for example, generating the DSU through the typical machining case of the SN and the input information of human–computer interaction. Modulator, simulating the function of the amygdala, provides a control directive, according to the preprocessing result, such as the mode of human interaction and the way of interrupt handling. Executor, which simulates the function of the frontal cortex, controls and finishes the knowledge-based processing, for example, matching the function requirements of the PSU and extracting manufacturing effects and typical structures from the EN. Metabase, which simulates the function of the posterior cortex, stores the meta knowledge of the PIKU, such as process contradiction units and manufacturing effects. The retrieval protocol can support the corresponding components to retrieve the knowledge properties of Metabase and to further process problems. In this way, according to the process problem-solving rules, solving process, and knowledge processing architecture, the machining process problems are expected to be solved heuristically and structured with the semantic knowledge network.

## 5. Knowledge-Driven Process Problem Solving for Micro-Turbine Machining

Based on the proposed approach, we have designed and implemented a prototype system for knowledge-driven heuristic machining process innovation, named MPI-KHDS, which is integrated as a subsystem on the general CAPI platform containing the basic tools of innovation knowledge management and application.

**Figure 6:**
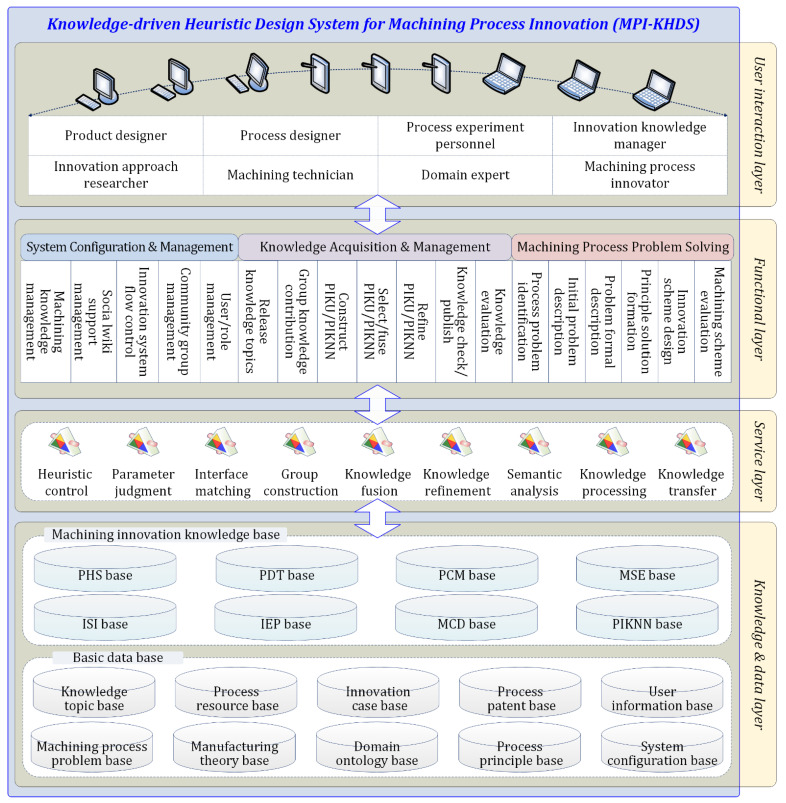
The system framework of MPI-KHDS.

The MPI-KHDS presents a four-layer-framework, as shown in Figure 6. The knowledge and data layer stores the basic source data of machining process innovation, and the formalized machining process innovation knowledge. The service layer provides access to the knowledge and data layer and supports various system background services of innovation knowledge acquisition and structured process problem solving. The functional layer provides the functions of system configuration and management, innovation knowledge acquisition and management, machining process problem solving and others. The user interaction layer provides a visual human–computer interaction interface for machining process innovation users, e.g., domain expert, machining process innovator, process designer, innovation approach researcher, and machining technician.

To support machining process problem solving effectively, we invited 9 domain experts, 20 machining technicians, and 30 graduate students to participate in the open knowledge acquisition of machining process innovation. After nearly 3 months of knowledge accumulation, the MPI-KHDS stored about 5000 pieces of refined process innovative knowledge in the micromachining field.

The KJ-66 micro-turbine is the core part of high-precision turbojet engines and is a typical micro-part with a complex thin-walled structure. However, there is no through hole in the turbine center. The positioning and clamping methods commonly used cannot be effectively applied to the machining process of this turbine; thus, the process problem relates to how to creatively implement the high-precision machining of the turbine using the existing manufacturing resources and process environment. In the following, we illustrate the application of knowledge-driven heuristic process problem solving for micro-turbine machining.

### 5.1. Process Problems Description of Micro-Turbine Machining

**Figure 7:**
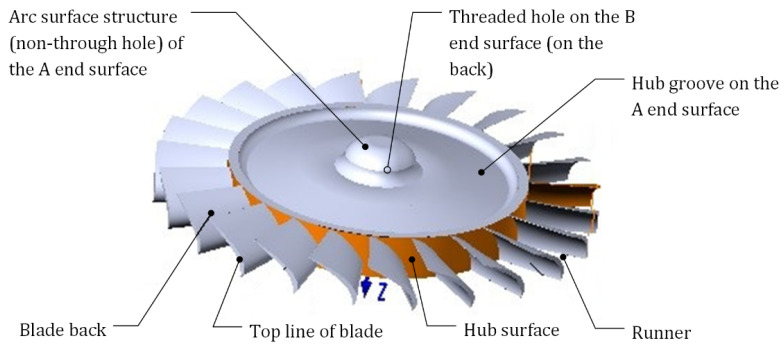
Structure diagram of a micro-turbine.

The KJ-66 micro-turbine is composed of a hub, several sets of blades evenly distributed along the circumference of the hub, and hub grooves on both ends. Its structure is shown in Figure 7. The micro-turbine is smaller in size than general turbines, and it requires higher machining accuracy. Its technological characteristics are as follows:

(1) The blade is a thin-walled part with poor process rigidity, more serious deformation, and higher processing accuracy requirements.
(2) As the size of the turbine is reduced, the diameter of the tool is also reduced, and so the rigidity of the tool becomes worse and is easier to break.
(3) The flow path between the blades is narrower and deeper, and the processing space is smaller. The relative swing of the tool during processing can easily cause cutting interference to the adjacent blades.
(4) The turbine center has no through-hole structure, which brings difficulties in turbine clamping.
(5) The thickness of the blade is variable. The thickness at the root of the blade is 1.1 mm, and the thickness at the tip of the blade is only 0.8 mm. The blade is thin and easily deformed. It is required to be free of burrs after machining.

The two ends of the turbine have bosses and arc surface structures with a height of approximately 4 mm. When machining the turbine blades, the four-jaw chuck of the CNC machine tool cannot directly and effectively clamp the two structures. In the process of micro-turbine blade machining, the chuck and core shaft are often used to achieve positioning and clamping, i.e., after the two end faces are machined, an axial through hole is drilled in the center of the micro-turbine, and the core shaft is clamped through the through-hole; thus, the turbine can be pressed on the chuck through the tightening force of the nut and the thread at one end of the core shaft. This method is suitable for the machining of a through-hole structure turbine. For a turbine without a through-hole, this method will destroy the structural integrity of the part itself. In addition, the positioning of the core shaft adopts a clearance fit, and the vibration generated during the machining process makes it difficult to ensure the accuracy of the blade. Blade machining is key to the manufacturing of the micro-turbine, so reliable clamping of the workpiece ensures accuracy in its size and shape.

According to conventional thinking, one method to solve the above problems is to change the structure of the turbine itself (e.g., drilling a central hole) to make it more convenient for clamping; however, this method will damage the structural integrity of the part. The alternative option is to replace the four-jaw chuck of the universal fixture and use more complex tooling and fixtures. In order to solve this process problem, under the existing processing conditions and without damaging the part structure or introducing too complex toolings and fixtures, we applied the acquired knowledge of the MPI-KHDS system to carry out process problem solving and innovation scheme design.

### 5.2. Knowledge-Inspired Process Innovation Scheme Design and Machining Experiment

**Figure 8:**
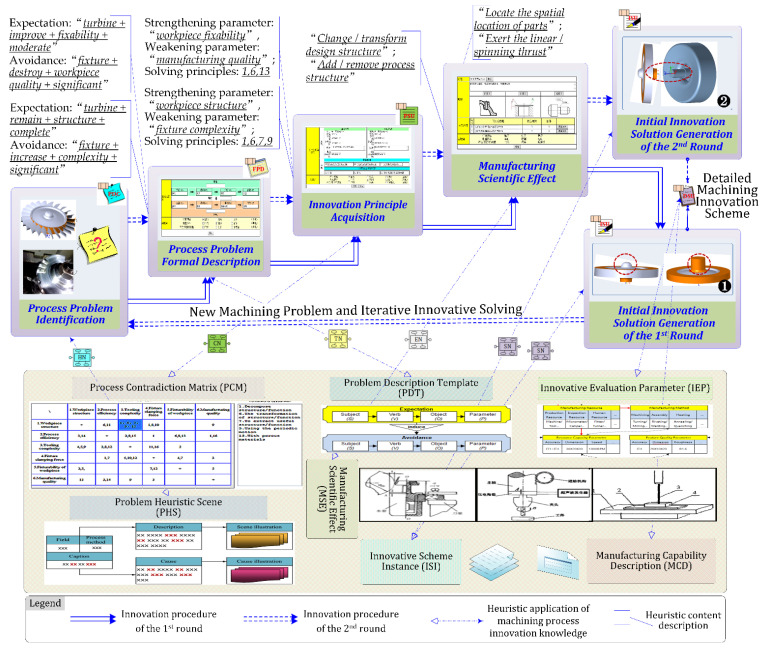
Knowledge-driven heuristic innovation scheme design for micro-turbine machining.

By using the knowledge-inspired innovation guidance of the MPI-KHDS system, the process designer can describe the process problem of turbine machining in detail, and gradually obtain innovation principles and solutions, as shown in Figure 8.

Through process problem identification, with the support of HNs, we define the first process problem to be solved as “how to clamp the turbine so that the blade can be machined with high quality.” The designer can formalize the process problem with the support of TNs, and output the FPD in an SVOP format (subject + verb + object + parameter) as: expectation: “turbine + remain + structure + complete”; avoidance: “fixture + increase + complexity + significant.” Subsequently, the system will automatically match the corresponding knowledge units in the process contradiction matrix, according to the formal input content and output the corresponding innovation principles. In this problem-solving process, we obtain the following PSU with help from CNs: strengthening parameter: “workpiece structure,” weakening parameter: “fixture complexity”; solving principles: 1, 6, 7, 9. The corresponding solution principles are: 1. split structure/function; 6. convert structure form/function; 7. extract useful structure/function; 9. use periodic actions. Through analysis from designers, it is found that the two innovation principles (6, 7) have a greater heuristic effect on solving process problems. The heuristic result that can be obtained is that, in addition to the blades of the micro-turbine, the available parts are the arc bosses on the A end face and the threaded hole structure on the B end face, which can be transformed into useful structures or functions during machining. Thus, the designer can check the heuristic solution principle (6, 7) and select the corresponding industry field, manufacturing technology, and product parts and other keywords. Further, the system will provide the corresponding manufacturing scientific effect (ENs in this step are “change/transform design structure”; “add/remove process structure”) to help the designer clarify their innovation ideas. Subsequently, the process innovation knowledge network of the system can be generally matched to the corresponding innovative scheme instances. As there is no ISI matched for this problem, the system will turn to the manual solution here. Based on the solving principles and manufacturing scientific effects obtained, we retrieve the initial innovation solution as follows: the arc surface structure of the micro-turbine A end face can be transformed into a form that can provide clamping, i.e., change its form and function in the machining environment to facilitate the clamping of the four-jaw chuck. After the turbine blade is machined, the process boss can be machined into a circular arc surface structure.

When the turbine blades are processed, there is a reserved process boss on the A end face, which needs to be machined into a circular arc surface structure. Considering that the finishing machining of the blades has been completed, and the turbine blades are thin-walled structures, it is easy to crush the finished blade and even cause blade deformation, if the turbine is clamped using the pressing plate. However, if the turbine blade is not clamped, it will not be possible to accurately cut off the process boss and machine the arc surface structure. Thus, the new process problem is how to clamp the micro-turbine to mill the process boss when the blades have finished being machined. The machining quality of the turbine blades and the clamping capability of the turbines constitute a contradiction that requires iterative innovative problem solving.

According to the above process problem analysis procedure, the second-round innovation principles of problem solving are obtained: strengthening parameter: “workpiece fixability,” weakening parameter: “manufacturing quality”; solving principles: 1, 6, 13. Through analysis, it is considered that principle 6 (convert structure form/function) and principle 13 (use its own structure/function) provide significant contributions to this process problem solving. In the first round of process problem analysis, we are inspired by principle 6 by which the structure or function of the arc boss or threaded hole on the two ends can be transformed. However, the process boss on the A end face improves the machining quality of the blade, but causes the current problem. Therefore, the use of the form or function of the threaded hole structure on the B end face may become a problem-solving direction. In combination with the inspiration of using its own structure/function of principle 13, a solution to this problem can be that the threaded hole structure on the B end surface for the final product assembly can be used in workpiece clamping and positioning. Process innovators can consider making full use of the counterbore and thread of the workpiece itself and design a fixture with threads to connect with it, so that the process boss can be milled into the final shape without affecting the surface quality and dimensional precision of the micro-turbine blades.

**Figure 9:**
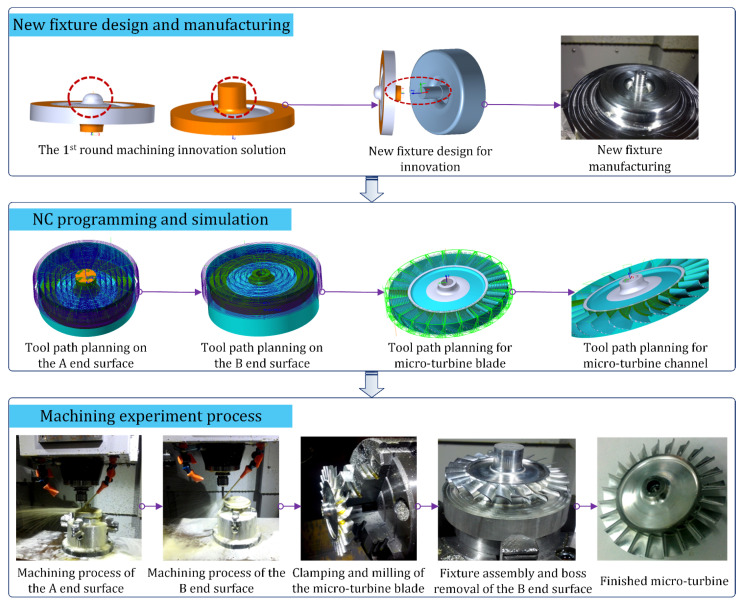
Machining process innovation experiment of the micro-turbine.

On the basis of the solving principles for the above two rounds of machining process problems, a new fixture was manufactured and a detailed micro-turbine clamping and machining scheme was designed. After NC tool path planning for the key machining position of the turbine was simulated using ES-Surfmill6.0 software, we carried out the micro-turbine machining experiment using a Smart CNC500 machine tool. In Figure 9, it can be seen that, compared with the original mandrel positioning scheme, the clamping and positioning of the process innovation scheme, with the new fixture, are more reliable, while vibration in the machining process is less, the surface quality of the blade is obviously improved, and there is no tool interference during the machining process.

## 6. Conclusions

Process problem solving in micromachining, such as unreliable clamping, thin-walled deformation, and low surface quality, is vital to ensure quality assurance of micropart production. Knowledge plays an intrinsic key role in the procedure of manufacturing process innovation. In this paper, a knowledge network-driven systematic scheme for micromachining process innovation is presented. The main contributions of this study can be summarized as:

- By analyzing the knowledge requirements of computer-aided machining process innovation, several types of MPIK units and the corresponding knowledge network are formally represented. An open multi-source MPIK acquisition and management approach based on collective intelligence is introduced.
- In considering the specific role of formal knowledge in human–computer interaction innovation, a knowledge network-driven structured problem-solving and heuristic innovation design procedure for the machining process is presented that can support process planners in reducing inherent mindsets and individual knowledge limitations and facilitate knowledge-based heuristic innovation.
- The specific micromachining process problem-solving and innovation design for a micro-turbine, without a through-hole, is completed using the innovation support prototype system, MPI-KHDS. The machining experiment shows that the machining quality of the micro-turbine, with the innovation scheme, is significantly improved.

## Figures and Tables

**Table 1:** Concept and function of each type of MPIK.

Type	Concept	Function
Problem Heuristic Scene (PHS)	Abstract and formalized phenomenon description of typical process problems to facilitate the cause analysis of problem scenes	To stimulate technicians to associate their tacit knowledge in order to recognize and analyze problems effectively and correctly
Problem Description Template (PDT)	Formalized representation framework according to the essential structure of machining process problem	Providing constraints for the formal description of process problems to ensure the clarity of problem expression and the understandability
Process Contradiction Matrix (PCM)	The relationships between technical parameters, contradictions, and process innovation principles	Providing the solving direction and principal solution for the structural conflict resolution of process problems
Manufacturing Scientific Effect (MSE)	Manufacturing-oriented and multidisciplinary basic scientific effects	Providing the basic technical framework and scientific effects for preliminary innovative solution design
Innovative Scheme Instance (ISI)	Formalized, practical, and successful schemes for typical process problems	Providing technical implementation reference solutions to support the detailed innovative scheme design
Innovative Evaluation Parameter (IEP)	Evolution path parameters and lifecycle phase parameters of all technical systems	Providing the quantitative evaluation parameters to identify the innovativeness grade of innovative solution
Manufacturing Capability Description (MCD)	Formalized description of manufacturing capability for the specific enterprise	Evaluating the manufacturing feasibility of the innovative scheme in the firm-specific manufacturing environment

**Table 2:** The basic ontologies for machining process innovation.

Type	Ontology Contents
Process Data Ontology	Initial Problem Description (IPD)
	Problem Description with Cause (PDC)
	Formalized Problem Description (FPD)
	Principle Solution (PSU)
	Initial Solution (ISU)
	Detailed Solution (DSU)
	Solution Innovativeness (SIV)
	Solution Manufacturability (SMF)
Knowledge Ontology	Heuristic Neuron (HN)
	Template Neuron (TN)
	Contradiction Neuron (CN)
	Effect Neuron (EN)
	Solution Neuron (SN)
	Innovativeness Neuron (IN)
	Manufacturability Neuron (MN)

**Table 3:** The basic rules of knowledge-based heuristic machining process problem solving.

Rule	Description
Interface Matching	Considering that the semantic relations and processing rules among various types of machining PIKUs are explicit, the forward propagation mode is adopted to transfer the parameters without feedback learning. Through the output interface, the current PIKU will select all subsequent PIKUs that can accept their output parameter types and transfer the output results to the input interface of subsequent PIKUs.
Parameter Judgment	There are two methods for judging the effectiveness of input parameters. The first is to determine automatically whether the parameter is effective, according to the processing rules, and the second is to manually intervene. In both ways, as long as the parameters are ineffective, the current machining PIKU will interrupt its knowledge transfer process.
Knowledge Processing	There are two ways to process the input parameters. One way is to automatically process by PIKUs, based on the knowledge properties and processing rules, while the other way requires manual intervention and to output the processing results.
Manual Solving	If there are no suitable machining PIKUs to use at a solving step of process innovation, then the solution process will be transferred to manual solving. Subsequently, the result of manual solving will be transferred to the next-level PIKUs to continue the solving process.
